# Preparation and Characterization of Alginate Hydrogel Fibers Reinforced by Cotton for Biomedical Applications

**DOI:** 10.3390/polym14214707

**Published:** 2022-11-03

**Authors:** Farooq Azam, Faheem Ahmad, Sheraz Ahmad, Muhammad Sohail Zafar, Zeynep Ulker

**Affiliations:** 1 School of Engineering & Technology, National Textile University, Faisalabad 37610, Pakistan; 2 Department of Restorative Dentistry, College of Dentistry, Taibah University, Al Madinah, Al Munawwarah 41311, Saudi Arabia; 3 Department of Dental Materials, Islamic International Dental College, Riphah International University, Islamabad 44000, Pakistan; 4 School of Pharmacy, Altinbas University, Istanbul 34147, Turkey

**Keywords:** hydrogel fiber, alginate, mechanical strength, biomedical, reinforcement and cotton fibers

## Abstract

In this study, cotton-reinforced alginate hydrogel fibers were successfully synthesized using the wet spinning technique to improve hydrogel fibers’ mechanical strength and durability. Structural, chemical, and mechanical properties of the prepared fibers were characterized using scanning electron microscopy, Fourier transform infrared spectroscopy, X-ray Diffraction, differential scanning calorimeter, and single fiber strength tester. Based on the results obtained from fourier transform infrared spectroscopy and x-ray Diffraction, cotton fibers have been successfully incorporated into the structure of the hydrogel fibers. It was seen from the differential scanning calorimeter results that the incorporation of fibers in the structure even enhanced the thermal stability of the fiber and is viable to be implanted in the human body. Cotton reinforcement in alginate hydrogel fibers increases the modulus up to 56.45 MPa providing significant stiffness and toughness for the hydrogel composite fiber. The tenacity of the fibers increased by increasing the concentration of alginate from 2.1 cN/Tex (1% *w*/*v*) to 8.16 cN/Tex (1.5% *w*/*v*). Fiber strength increased by 26.75% and water absorbance increased by 120% by incorporating (10% *w*/*w*) cotton fibers into the fibrous structure. It was concluded that these cotton-reinforced alginate hydrogel fibers have improved mechanical properties and liquid absorption properties suitable for use in various biomedical applications.

## 1. Introduction

Hydrogels are three-dimensional network structures made up primarily of natural or synthetic hydrophilic compounds. They are capable of absorbing a substantial amount of fluids and become swelled [1,2]. Hydrogels are widely used in bone tissue engineering [3], wound healing [4], drug delivery [5], cartilage tissue engineering [6], protein delivery, and antibiotics [7,8]. The composition of hydrogels should be compatible with the physical, biological, and mechanical requirements of the specific application in various fields [9,10]. The strength of hydrogel structures is mainly dependent upon the crosslinking density (physical or chemical) [11]. Hydrogels have been produced from various synthetic and natural materials. Chitosan [12], alginate [13], and poly(ethylene glycol) fibrinogen [14] produce chemical networks in hydrogels while aminated hyaluronic acid [15] and collagen [16] can produce physical networks in hydrogels. Among all available materials, alginate is an excellent crosslinking material obtained from seaweeds and is widely used in food processing, pharmaceuticals [17], paper formation, and textiles [18]. Alginate is biocompatible and has very low toxicity making it suitable material for biomedical applications [19]. In addition, alginate hydrogel fiber can be synthesized easily by the conventional wet spinning technique on a commercial scale.

Despite being soft and biocompatible, hydrogels are brittle and weak, which restricts their use in a variety of fields. Hydrogels are primarily used in biomedical applications [20]. As a result, to enhance the mechanical properties of the hydrogels, the textile fabric is used to create hydrogel composites [21]. It has been reported that numerous studies have been conducted on textile fabric-reinforced hydrogel composites for use as wound dressings. Chitosan was found to improve the antibacterial activity of the fabric against both Gram-positive and Gram-negative bacteria when it was used to develop an antibacterial hydrogel composite reinforced with cotton fabric in a study [22]. In another study, Hubbard et al. [23] synthesized hydrogel reinforced with elastomer glass fiber to enhance the mechanical properties of the hydrogels. They found improved interfacial bonding (1000 Nm^−1^) between the fabric and hydrogel.

Both hydrogels and textile fabrics have their properties improved by fabric-reinforced composite. The hydrogel matrix in the composite is essential, and the fabric gives the composites their mechanical strength. Researchers are still working to improve the drapability of hydrogel composites even though fabric reinforcement increases the mechanical strength of hydrogels. Additionally, pure hydrogel fibers are unable to form three-dimensional network structures that can contain infiltration [24]. Thus, various researchers have worked to combine the advantages of electrospun fibers and hydrogel to make a functional composite system [25,26].

The addition of fibers in the hydrogels to make fiber-reinforced hydrogel composite significantly improved the mechanical properties of the hydrogels due to the fiber’s structural support [27]. Regev et al. [28] developed electrospun fibers reinforced hydrogel composites using bovine serum albumin fibers as reinforcement and gelatin as hydrogels. They found that the addition of fibers improved the modulus of elasticity of hydrogel and decrease the gelation time. Tonsomboon and Oyen [29] reinforced alginate hydrogels with gelatin fibers to enhance the rigidity and tensile modulus of the hydrogels.

Natural fibers are the potential candidates to be used as reinforcement in the composites due to their unique properties such as low density, high modulus, and tenacity, nontoxic, biodegradable, and nonabrasive characteristics [30,31]. In addition, these materials are cost-effective and environmentally friendly and, therefore, are continuously used in hydrogels and polymer composites for various biomedical applications. Synthetic fibers are nonrenewable, nonbiodegradable, and nonrecyclable and hence, cannot be used as reinforcement for biomedical applications [32]. Cotton is one of the most versatile natural fibers that provide biodegradability, high tensile strength, durability, and high-water absorbency. Cotton fibers have been used in many external and internal biomedical applications such as surgical clothing, wound dressing, drug delivery, and tissue engineering [33]. Alginate is proven as a biodegradable and nontoxic material when administrated orally [34]. Furthermore, alginate implants showed no immune response and do not cause foreign body reactions, after three weeks of implantation in the peritoneal cavity of rodents [35,36]. Zhang et al. [37] fabricated scaffolds made up of cotton fabric reinforced PVA/alginate hydrogels. Fiber reinforcement in hydrogel structures is a promising method to improve the mechanical properties of tissue engineering scaffolds [38,39].

Most of the authors’ work in the previous studies is related to electrospun fibers hydrogel composites. To our knowledge, there is no study involving natural fiber-reinforced hydrogel composite fibers. Thus, the main purpose of this study was to develop cotton fibers reinforced hydrogel fibers with improved mechanical properties for the biomedical field.

## 2. Materials and Method

Sodium alginate and calcium chloride (CaCl_2_ anhydrous) were purchased from DAEJUNG Korea. Cotton fibers were provided by NATIONAL TEXTILE UNIVERSITY Faisalabad. Raw cotton was taken from the market and then it was pre-cleaned through mechanical action to remove the trash. The properties of the cotton fibers that have been used in this study are given in Table 1.

### 2.1. Preparation of Dope Solution

**Figure 1:**
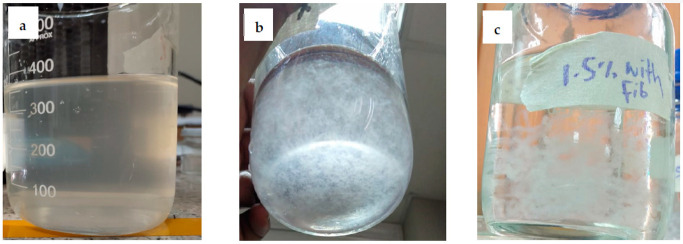
(**a**) Prepared alginate solution, (**b**) cotton fibers dispersion in alginate solution, (**c**) cotton reinforced alginate hydrogel fibers.

1% (*w*/*v*), 1.25% (*w*/*v*) and 1.5% (*w*/*v*) aqueous solutions of sodium alginate were prepared by stirring at 800 rpm on magnetic stirrer for 24 h at room temperature. Subsequently, Pre-cleaned cotton fibers were cut into 2 ± 0.5 mm lengths. Then, these short-length fibers (10% *w*/*w*) were dispersed in the prepared sodium alginate solution using a pneumatic stirrer. Prepared alginate solution, cotton fibers dispersion in alginate solution, and cotton reinforced alginate hydrogel fibers are given in Figure 1.

### 2.2. Fiber’s Production

**Figure 2:**
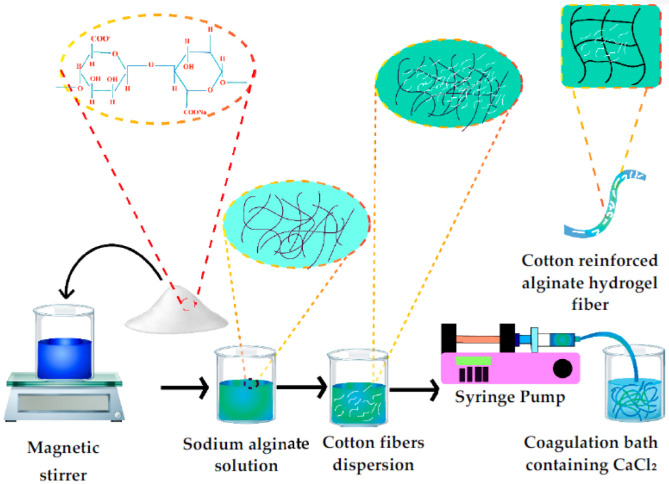
The schematic diagram for the synthesis of cotton-reinforced alginate hydrogel fibers.

The prepared dope solution of alginate containing cotton fibers was then poured into the dope tank. The dope solution was then pumped through a needle of 18 gauge into the coagulation bath having a 1% *w*/*v* solution of calcium chloride. The fibers then remained in a coagulation bath for 15 min for proper crosslinking of alginate molecules. Fibers were then removed from the coagulation tank, rinsed thoroughly with distilled water, and finally dried at room temperature. The schematic diagram to produce cotton fiber-reinforced hydrogel composite fibers is given in Figure 2.

### 2.3. Characterization of Fibers

#### 2.3.1. Tensile Strength

The tensile strength of the fibers was calculated using a single fiber strength tester (M250-2.5CT Testometric, Rochdale, England) in accordance with standard ASTM D3822. ASTM D 1776 standard was used to precondition the samples. The test for single fiber strength was performed with a 50 N load having a gauge length of 25 mm at a speed of 100 mm/min at room temperature and pressure. Five replications of each sample were taken, and the mean value was recorded.

#### 2.3.2. Water Absorption

The water absorbency of the fibers was investigated using the ASTM D570 standard. The sample was first dried in an oven for 24 h at 50 °C and then weighed (*W_d_*) the samples on analytical balance with least count 0.1 mg. The preconditioned samples were then placed in distilled water for 24 h at ambient temperature and pressure. After 24 h the samples were removed, patted dried, and then finally weighed (*W_w_*). After calculating the dry and wet weights of fibers the water absorbency was calculated using Equation (1).

(1) $$Water\;absorption\;\left(\frac{g}{g}\right)=\frac{Ww-Wd}{Wd}\times 100$$

#### 2.3.3. FTIR

FTIR (Fourier transform infrared spectroscopy) manufactured by PerkinElmer was used to characterize the functional groups of alginate and cotton fibers in transmission. Since the samples were in solid-state therefore firstly sample is placed on the top surface of the crystal. Then, placed the gripper plate on the sample and the pressure applied to the gripper plate was adjusted to ensure consistent contact is achieved between the crystal and the sample. Then, each sample was scanned from 4000 cm^−1^ to 600 cm^−1^.

#### 2.3.4. X-ray Diffraction

X-ray diffractometer XPert Pro manufactured by PANalytical was used to investigate the crystallographic information of hydrogel fibers. The data were collected at 2θ from 10–40° with a step size of 0.01°.

#### 2.3.5. Surface Morphology

The surface morphology of the fibers was investigated using scanning electron microscopy (SEM) and a light microscope. Since samples were non-conductive so samples were gold coated using a sputter coater before analysis.

#### 2.3.6. DSC

A differential scanning calorimeter (DSC 400, Perkin Elmer, Waltham, MA, USA) was used to investigate the thermal properties of the hydrogel fiber from 25 °C to 400 °C in an inert environment at the heating rate of 5 °C/min.

## 3. Results and Discussion

### 3.1. Surface Morphology

**Figure 3:**
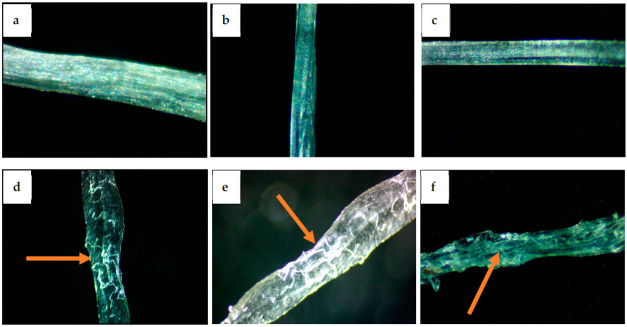
Microscopic images of Pure alginate Hydrogel fibers ((**a**): 1%, (**b**): 1.25%, (**c**): 1.5%) and cotton reinforced alginate hydrogel fibers ((**d**): 1%, (**e**): 1.25%, (**f**): 1.5%).

**Figure 4:**
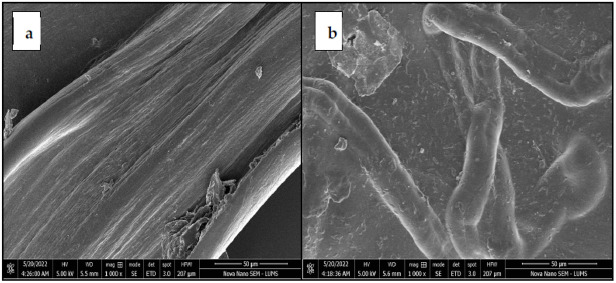
(**a**) SEM image for hydrogel fiber without reinforced fibers (**b**) SEM image for hydrogel with cotton fibers as reinforcement.

Microscopic images of pure alginate hydrogel fibers and cotton-reinforced alginate hydrogel fibers are shown in Figure 3. The reinforced cotton fibers can easily be seen and highlighted in Figure 3d–f. It can be seen from Figure 3a–c that the surface of the pure alginate hydrogel fibers is smooth as compared to the surface of the cotton reinforced alginate hydrogel fibers in Figure 3d–f. Cotton fibers are randomly oriented within the alginate fiber structure. This random orientation is confirmed by SEM images given in Figure 4b. The reason is that the presence of cotton fibers makes the surface uneven.

### 3.2. FTIR Spectrum

**Figure 5:**
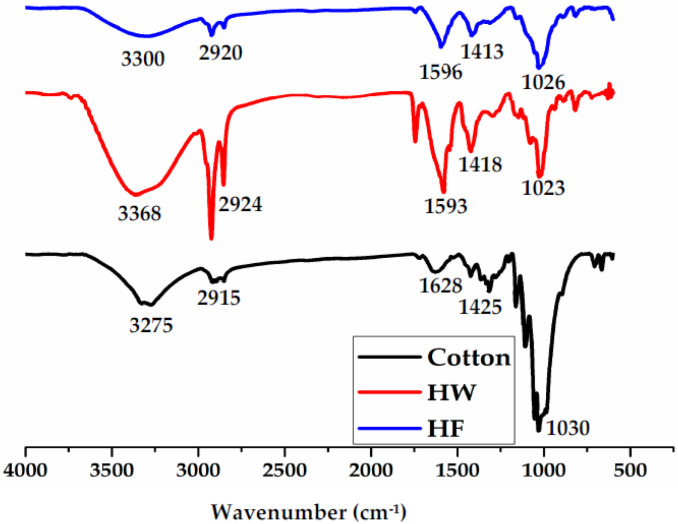
FTIR spectra of pure cotton fibers, Pure alginate hydrogel fibers (HW), and cotton reinforced alginate hydrogel fibers (HF).

The FTIR spectra of pure cotton fibers, pure alginate hydrogel fibers, and cotton reinforced alginate hydrogel fibers are given in Figure 5. A broader peak for pure cotton fibers from 3250 cm^−1^ to 3500 cm^−1^ represents the –OH group present in the cellulosic structure. Peaks around 2900 cm^−1^ and 1630 cm^−1^ correspond to C–H stretching and –CO stretching, respectively. The peak near 1020 cm^−1^ and 1417 cm^−1^ were attributed to the C–O stretching and C=C stretching, respectively. These all peaks confirm the structure of cotton fibers. The IR spectra of pure alginate hydrogel fiber showed a characteristic peak at around 1600 cm^−1^ which corresponds to the asymmetric stretch of COO– of carboxylic acid salt [40]. Another prominent peak can be seen around 3200 cm^−1^ attributed to the –OH group due to the presence of hydrogen bonding between the molecules. Two peaks near 1030 cm^−1^ and 1100 cm^−1^ correspond to the –CH stretching and –OH bending, respectively [41]. Based on the IR spectra of the cotton reinforced alginate hydrogel fibers, it is shown that the peaks of both alginate, as well as cotton fibers, can be seen in the highlighted area. The peak intensity decreases at near 3300 cm^−1^ and at near 2900 cm^−1^ which is due to the presence of cotton fibers in the alginate structure. The formation of intermolecular hydrogen bonding between cotton fibers and alginate hydrogel is attained due to the presence of hydroxyl groups and carbonyl groups that may decrease and shifts the intensities of the peaks [42,43].

### 3.3. Thermal Characterization

**Figure 6:**
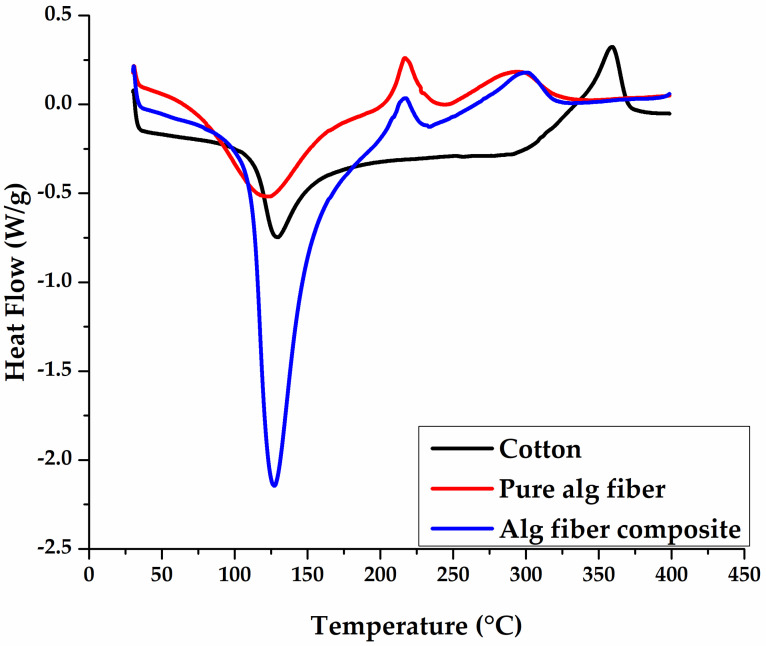
DSC thermograms of cotton, pure Hydrogel alginate fiber, and cotton reinforced alginate hydrogel fiber.

DSC thermogram of pure cotton fibers, pure alginate hydrogel fibers, and cotton reinforced alginate hydrogel fiber is given in Figure 6. The endothermic peak at 100–130 °C characterizes the loss of water and melting of wax including, lipids, fatty acids, and proteins present in cotton fiber [44,45]. The second exothermic peak at around 370 °C represents the decomposition or degradation of cotton followed by the formation of other volatile products [46]. Pure alginate hydrogel fiber shows an endothermic peak at around 100 °C that may be correlated to loosely bound water elimination linked to COO groups and melting of crystalline structure [47]. Two exothermic peaks at around 240 °C and 320 °C are correlated to the polymer degradation i-e pyrolysis reactions, respectively [48]. A slight peak shift towards high temperature and increase in peak intensity can be seen at around 130 °C for Cotton reinforced alginate hydrogel fiber. This phenomenon can be attributed to the intermolecular hydrogen bonding between the alginate molecules and cotton fibers due to the presence of –OH groups and carbonyl groups [42,45]. This curve shows that the presence of cotton fiber in alginate fibers causes little change in thermal behavior.

### 3.4. X-ray Diffraction

**Figure 7:**
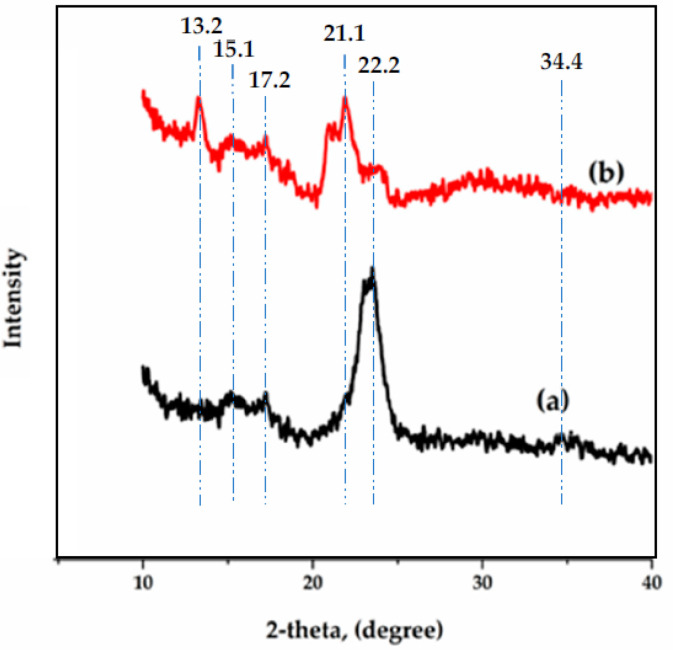
XRD Spectra of (**a**) pure cotton fibers (**b**) cotton fibers reinforced alginate hydrogel composite fiber.

X-ray spectra of pure cotton fibers and cotton fiber-reinforced alginate hydrogel composite are given in Figure 7. The X-ray diffraction pattern of cotton fibers which have semi-crystalline nature, as shown in Figure 7a. The strong peaks at 2θ = 15°, 17°, 22° and 34° represent the crystalline form of cellulose 1 [49]. Figure 4b shows the diffraction spectrum of cotton reinforced alginate hydrogel composite. The characteristic peaks at 2θ = 13° and 21° are associated with alginate [50] while peaks at 2θ = 15°, 17°, 22° and 34° show the cotton structure which confirms the cotton fiber reinforcement in alginate fibers. The peak intensity at 2θ = 15°, 17°, 22°, and 34° in Figure 7b decreases as compared to Figure 7a because 10 wt% cotton fibers were added to alginate fiber as reinforcement which enhances the amorphousness of the hydrogel polymers [43]. The peaks for cotton reinforced alginate hydrogel fiber are not very sharp which exhibits a semi-crystalline structure.

### 3.5. Mechanical Strength

**Figure 8:**
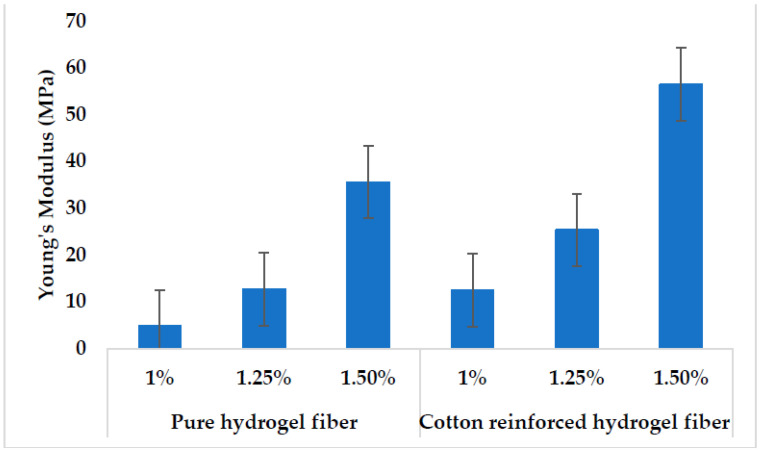
Young’s Modulus of pure alginate hydrogel fibers and cotton reinforced alginate hydrogel fibers.

**Figure 9:**
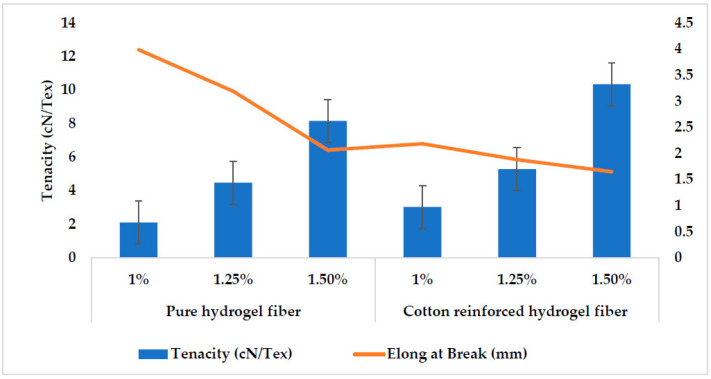
Tensile Strength of pure hydrogel fibers and cotton reinforced hydrogel fibers at different concentrations.

**Figure 10:**
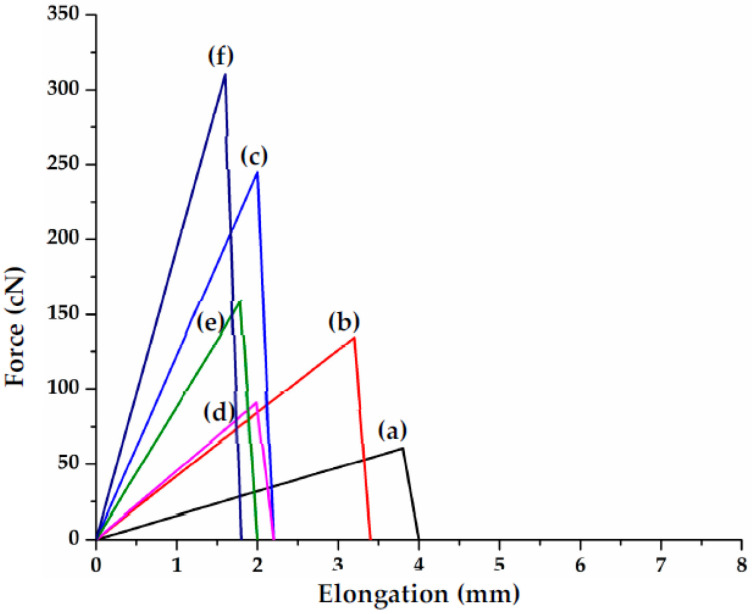
Stress–strain curve of pure alginate hydrogel fibers ((**a**): 1%, (**b**): 1.25%, (**c**): 1.5%) and cotton reinforced alginate hydrogel fibers ((**d**): 1%, (**e**): 1.25%, (**f**): 1.5%).

The mechanical characteristics of biomaterials play a significant role in tissue engineering because the stiffness of biomaterials is important in activating the intracellular signaling process [51]. Therefore, mechanically strong material is the requirement to be used in biomaterials applications such as scaffolds for tissue engineering. Various studies have been reported to improve the mechanical properties of soft tissue scaffolds, and they achieve modulus ranging from KPa to MPa [52]. In a study, silk fibers were used as reinforcement to improve the mechanical properties of gellan gum hydrogel, but could achieve a maximum of 0.4 MPa modulus [53]. In another study, hydrogel for tissue engineering scaffolds has been developed using sodium alginate, gelatin, and soy protein powder and could get a 256.7 kPa modulus [54]. Young’s modulus for pure alginate hydrogel fibers and cotton reinforced alginate hydrogel fibers is given in Figure 8. Cotton reinforcement in alginate hydrogel fibers increases the modulus up to 56.45 MPa as shown in Figure 8. Pure alginate hydrogel fiber with 1 wt% shows the least value for modulus (4.74 MPa). Tensile strength and elongation at break for the pure alginate fibers and cotton reinforced alginate hydrogel fibers with various concentrations are given in Figure 9 and Figure 10, respectively. As expected, the tensile strength of the cotton reinforced alginate hydrogel fiber depends upon the cotton fibers present within the alginate hydrogel fiber. The tensile strength and modulus of the cotton reinforced alginate hydrogel fiber are greater than pure alginate hydrogel fibers. This might be due to the interaction between the alginate molecules and cotton fibers because both have hydroxyl groups in their structure. Therefore, interfacial hydrogen bonding enhanced the internal structure and was tough enough to hold greater stress. Such a physical structure that is crosslinked internally can transmit the force and dissipate the energy partially by bond breakage and then recombine [55]. The results also showed that by increasing the concentration of alginate, the tensile strength and modulus of the fibers also increase. 1.5% concentration of alginate showed the highest value of tensile strength for both pure alginate fibers (8.16 cN/tex) and cotton reinforced alginate hydrogel fibers (10.343 cN/tex) while 1% concentration showed the least value (2.1 cN/tex). The possible reason is that by increasing the concentration of alginate, there will be more molecules crosslinked per unit area and hence tenacity will increase. The line chart in Figure 9 shows elongation at break values for all the samples, which shows an overall decreasing trend by increasing the alginate concentration. The possible reason is that by increasing the concentration of alginate more cross-linking of molecules will occur and hence the rigid structure will develop as evident from the stress–strain curve in Figure 10. Furthermore, by adding the cotton fibers as reinforcement within the fiber structure, elongation at break is further reduced because of the interaction of cotton fibers with the alginate structure.

### 3.6. Water Absorbency

High water absorbency is the primary characteristic of hydrogel fibers. One of the important characteristics of scaffolds is their water content and water-retaining capability. It is important to absorb the exudates from a wound to promote the healing process by controlling cellular dehydration and promoting angiogenesis and collagen synthesis [56]. The suitable moisture control enhances the healing rate, reduces pain, and provide protection for wound from infection [57]. Exudates from wounds separate the tissue layers resulting the slower healing. Therefore, water absorption is an important characteristic for hydrogel fibers to be used as scaffolds for various biomaterials applications.

**Figure 11:**
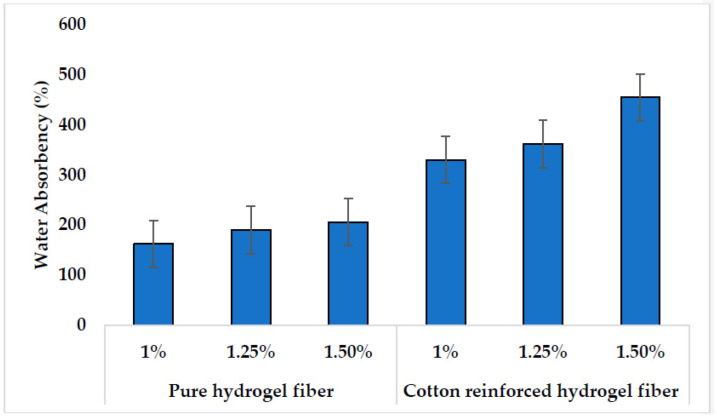
Water absorption property of pure hydrogel fiber and cotton reinforced hydrogel fiber at different concentrations.

The water absorption (%) property of the pure alginate hydrogel fibers and cotton reinforced alginate hydrogel fibers with different alginate concentrations is given in Figure 11. Figure 11 shows high water absorbency for all the concentrations of alginate fibers with or without reinforcement. Water absorbency increases by increasing the concentration of alginate as 1.5% concentration have a high value of water absorption and 1% has the least. On the other hand, the water absorption of cotton reinforced alginate hydrogel fibers is greater than the pure alginate hydrogel fibers. The reason is that the reinforcement is cotton fiber, which is also hydrophilic and capable of absorbing water. The water absorbency of pure cotton fibers is 300%. Therefore, the addition of cotton in hydrogel fibers raised the water absorbency of cotton-reinforced alginate hydrogel fiber. Consequently, adding the reinforcement to the alginate fibers increases the water absorption of the fiber.

## 4. Conclusions

The alginate hydrogel fibers reinforced with cotton fibers with improved strength were synthesized using the wet spinning technique and their properties were compared with pure alginate hydrogel fibers. Microscopic images indicated that the surface of the fiber became rough with the addition of cotton fibers. The thermal stability of the fibers increases with the incorporation of cotton fibers as reinforcement in the hydrogel fibrous structure. The developed alginate hydrogel composite fiber exhibited substantial improvement in mechanical properties compared with neat alginate hydrogel fibers. The modulus of the neat alginate hydrogel fibers increases from 4.74 MPa to 35.58 MPa by increasing the initial concentration of alginate fibers from 1% *w*/*v* to 1.5% *w*/*v*. The prepared cotton-reinforced alginate hydrogel fibers with biocompatibility and high-water absorbency (120%) exhibited extremely high modulus (56.45 MPa) and tenacity (10.34 cN/tex). Therefore, alginate hydrogel fibers with improved strength have been successfully synthesized by incorporating the cotton fibers as reinforcement within the alginate hydrogel fibrous structure. These fibers can be used to develop scaffolding systems with better mechanical and medicinal properties.

## Figures and Tables

**Table 1:** Properties of cotton fibers.

Moisture Content (%)	Micronaire Value	Fiber Length (mm)	Uniformity Index (%)	Strength (g/tex)	Elongation (%)
7.5	4.47	26.72	81.8	28	5.4

## Data Availability

Not applicable.
